# Paraquat Degradation From Contaminated Environments: Current Achievements and Perspectives

**DOI:** 10.3389/fmicb.2019.01754

**Published:** 2019-08-02

**Authors:** Yaohua Huang, Hui Zhan, Pankaj Bhatt, Shaohua Chen

**Affiliations:** State Key Laboratory for Conservation and Utilization of Subtropical Agro-Bioresources, Integrative Microbiology Research Centre, Guangdong Province Key Laboratory of Microbial Signals and Disease Control, South China Agricultural University, Guangzhou, China

**Keywords:** paraquat, bioremediation, microbial degradation, degradation pathways, oxidation

## Abstract

Paraquat herbicide has served over five decades to control annual and perennial weeds. Despite agricultural benefits, its toxicity to terrestrial and aquatic environments raises serious concerns. Paraquat cannot rapidly degrade in the environment and is adsorbed in clay lattices that require urgent environmental remediation. Advanced oxidation processes (AOPs) and bioaugmentation techniques have been developed for this purpose. Among various techniques, bioremediation is a cost-effective and eco-friendly approach for pesticide-polluted soils. Though several paraquat-degrading microorganisms have been isolated and characterized, studies about degradation pathways, related functional enzymes and genes are indispensable. This review encircles paraquat removal from contaminated environments through adsorption, photocatalyst degradation, AOPs and microbial degradation. To provide in-depth knowledge, the potential role of paraquat degrading microorganisms in contaminated environments is described as well.

## Introduction

Paraquat or methyl violet(1,1′-dimethyl-4,4′-bipyridinium dichloride) is a broad-spectrum cationic contact herbicide that is widely used in more than 100 countries (Rashidipour et al., 2019). Paraquat is applied against annual or perennial weeds of cotton, rice, soybean, and cocoa (Paraquat Information Center, 2018). However, because of high environmental and human toxicity, it was banned in some countries including Austria, South Korea and the European Union (Cha et al., 2016; Bang et al., 2017; Verssimo et al., 2017). China banned paraquat aqueous solution in 2016 but the pesticide is still marketed under other formulations.

Paraquat was synthesized in 1882 but its role as a weedicide was discovered in 1955 and commercialized in 1962 by Imperial Chemical Industries (ICI or Syngenta) (Alexander, 1999). Low-cost, efficient weed elimination and a unique mechanism made it popular for massive applications. Paraquat deviates electron flow from photosystem that inhibits reduction of oxidized nicotinamide adenine dinucleotide phosphate (NADP^+^) during photosynthesis to produce PQ^+^ (Setif, 2015). Paraquat specifically targets photosynthesizing green plant parts where PQ^+^ is re-oxidized by the O_2_ produced in chloroplasts. During the re-oxidization, lethal superoxide radical (O^+^) is generated and its subsequent oxidation results in cell death (Reczek et al., 2017).

Paraquat is comparatively safe for soil microorganisms and plant roots, but its long-term exposure results in harmful biomagnification in humans and mammals (Frimpong et al., 2018). Extensive paraquat applications lead to widespread residues in soil surface and aquatic environments that ultimately enter the food chain (Pateiro-Moure et al., 2009).

**FIGURE 1:**
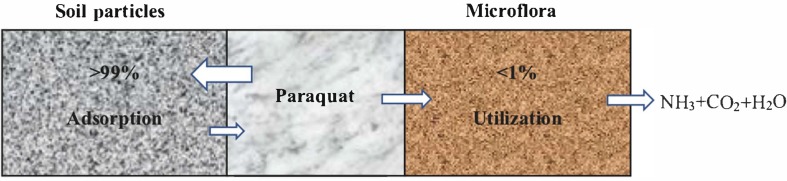
Equilibrium dynamics of soil and microorganisms for paraquat.

Depending upon the texture and composition, soil particles immediately adsorb paraquat (Singh and Singh, 2016). Rashidzadeh et al. (2017) described better paraquat adsorption in montmorillonite as compared to clinoptilolite clay and adsorption in clay is stronger than sandy soil (Amondham et al., 2006). Microorganisms can only utilize and degrade less than 1% of paraquat in soil particles (Figure 1) (Roberts et al., 2002), and its half-life can be up to 3∼6.6 years (Hance et al., 1980; Pateiro-Moure et al., 2009). Alexander (1999) concluded that microorganisms could completely degrade soil paraquat in 6 years. Such a prolonged half-life causes serious impact on humans and other mammals.

During the past few decades, paraquat poisoning has been reported on a global scale. According to the World Health Organization (WHO), the minimum lethal dose of concentrated paraquat in humans is 35 mg/kg (Tsai, 2013). Paraquat can cause neurological damage and dysfunctional kidneys and liver in humans and animals. In severe cases, fatalities can occur due to irreversible pulmonary fibrosis, inflammation, and respiratory failure (Blanco-Ayala et al., 2014; Shadnia et al., 2018). The paraquat toxicity mechanism is based on redox cycle and intercellular oxidative stress (Dinis-Oliveira et al., 2008). Flechel et al. (2018) revealed that out of 26 patients who ingested paraquat at a median intake of 103 mg/kg, only six survived after 36 h of emergency treatment. A study by Elenga et al. (2018) revealed higher adult mortality (65%) as compared to children (22%) due to difference in ingested amounts. In addition, paraquat is also associated with Parkinson’s disease (Bastias-Candia et al., 2019; Tamano et al., 2019). These studies indicate the severe toxicity of paraquat and its potential damage to mammalian cells.

Considering the hazards of paraquat residues on the environment and humans, it is necessary to study paraquat-degrading microorganisms. Microbial degradation is a significant pathway for paraquat breakdown (Mercurio et al., 2014; Wang et al., 2016) and various microorganisms including fungi, bacteria and yeast, have been reported for effective paraquat degradation (Wu et al., 2013; Bai et al., 2014). Anti-oxidative enzyme superoxide dismutase (SOD) contributes toward paraquat tolerance by removing superoxides from living cells, produced during paraquat toxicity (Dos Santos and Silva, 2015). Hoshina et al. (2018) indicated that catalase (CAT) could reduce paraquat cytotoxicity by increasing 4-phenylbutyrate. However, the literature lacks reports on microbial degradation pathways of paraquat, and no study has been reported about any functional gene of paraquat degradation. Here we aim to summarize microbial and physicochemical paraquat degradation methods and pathways, and analyze the potential of bioremediation in paraquat-contaminated environments. This review will increase the understanding about paraquat-contaminated sites and possible solutions through microbial applications.

## Physicochemical Methods for Paraquat Degradation

Paraquat herbicide is widely used in agriculture and silviculture; however, increasing attention is being paid to its soil residues. Currently, adsorption and degradation are the two main methods to remove/reduce paraquat from aquatic environment. Previous studies concluded that the adsorption of paraquat mainly depends on activated carbon (Sieliechi and Thue, 2015), activated bleaching soil (Tsai et al., 2004), modified zeolite (Pukcothanung et al., 2018), montmorillonite (Gu et al., 2015) and organoclay (Guegan et al., 2015; Keawkumay et al., 2017). On the other hand, physicochemical paraquat degradation methods depend on titanium dioxide, ozone, ultraviolet radiation and various advanced oxidation processes (AOPs) (Hamad et al., 2016; Gao et al., 2017; Javier et al., 2017).

A photocatalyst titanium dioxide has emerged as a promising degradation pathway for pesticide pollution treatment because of its low price, high efficiency and non-toxic properties (EI Madani et al., 2015; Phuinthiang and Kajitvichyanukul, 2019). To the best of our knowledge, the wavelength of incident radiation required to activate photocatalysis is related to bandgap energy of semiconductor materials, and larger bandgap energy requires shorter radiation wavelength (Liu et al., 2010). Titanium dioxide has a bandgap energy of 3.2 eV that requires ultraviolet light for activation (De Souza and Corio, 2013). Cantavenera et al. (2007) showed that the complete photocatalytic mineralization of paraquat (20 mg/L) was achieved after 3 h of irradiation by 0.4 g/L TiO_2_ at pH 5.8. According to Badli et al. (2016), similar conditions resulted in only 9.08% paraquat removal in the absence of photocatalysis and increased to 84.41% after the addition of ZrO_2_/TiO_2_ (20:80) at 0.3 g/L. This indicates that paraquat degradation necessarily requires oxygen, catalyst and UV-light.

Sorolla et al. (2012) reported 71% paraquat degradation by 2 wt.% Cu-TiO_2_/SBA-15 under visible light in 8 h that decreased to 67% at 5 wt.% Cu-TiO_2_/SBA-15. Scattering effect in suspension by excessive photocatalyst hinders light photons from entering the reaction mixture and reduces its paraquat degradation capability (Bensaadi et al., 2014). Kanchanatip et al. (2011) demonstrated that TiO_2_ along with vanadium and fullerene (C_60_) degraded 70% of paraquat under visible light after 4 h. Liu et al. (2014) evaluated photocatalytic degradation activities of HPW/MCM-48 against paraquat by loading the photocatalyst phosphotungstic acid H3PW12O40 (HPW) to molecular sieve MCM-48 through impregnation method under UV radiation (365 nm). Results showed that 63.79% paraquat (50 mL, 10 mg/L) was degraded by 20 mg HPW/MCM-48 catalyst after 14 h of UV irradiation whereas only 5% paraquat degradation was noted in the blank group.

In addition to the above-mentioned paraquat degradation techniques, other methods have also been reported. Fernandes et al. (2017) described new magnetic nanosorbents, composed of magnetite cores functionalized with bio-hybrid siliceous shells that can be used to uptake paraquat from water. Biopolymer k-carrageenan induction into the siliceous shells significantly increased its paraquat adsorption capacity at 257 mg/g. Desipio et al. (2018) proposed carbon nitride system as a catalyst to remove paraquat from water. Photocatalytic decomposition of paraquat solely by carbon nitride under visible light was negligible, but the addition of hydrogen peroxide in small amounts remarkably enhanced its paraquat degrading efficiency (70%) within 10 h.

Recently, various AOPs have emerged for the treatment of industrial or agricultural paraquat-contaminated wastewater. The principal mechanism of contaminant degradation through AOPs is based on the release of a highly reactive non-specific oxidant hydroxyl radical (OH) that dissociates organic molecules in water (Vilhunen and Sillanpaa, 2010; Rosman et al., 2018). Hydroxyl radical breaks larger molecules into smaller fragments that eventually mineralize to harmless products (Ong et al., 2014). Hydroxyl radical acts as a nucleophile during paraquat degradation. Treatment of wastewater by AOPs include UV/H_2_O_2_, ultraviolet, Fenton, photoelectro-Fenton, ozonation, photochemical, and electrochemical oxidation (Khongthon et al., 2016; Khataee et al., 2017). Dhaouadi and Adhoum (2009) reported photoelectric-Fenton and electro-Fenton as the most efficient treatments to remove paraquat from aqueous acidic solution at pH 3.0. Addition of 0.2 mM Fe^2+^ to the water containing 20 mg/L paraquat decreases its oxygen requirement by 97 and 94%, respectively. Similarly, Ye and Lemley (2008) investigated paraquat degradation in clay slurry through AOPs and found that strong adsorption of paraquat in clay interlayers protects the herbicide from hydroxyl radicals. The major disadvantage of physicochemical methods is their failure to control photo-catalysis conditions *in situ* remediation and that they are not cost-effective (Arora et al., 2018; Zhan et al., 2018a).

## Possible Pathways of Physicochemical Paraquat Degradation

**FIGURE 2:**
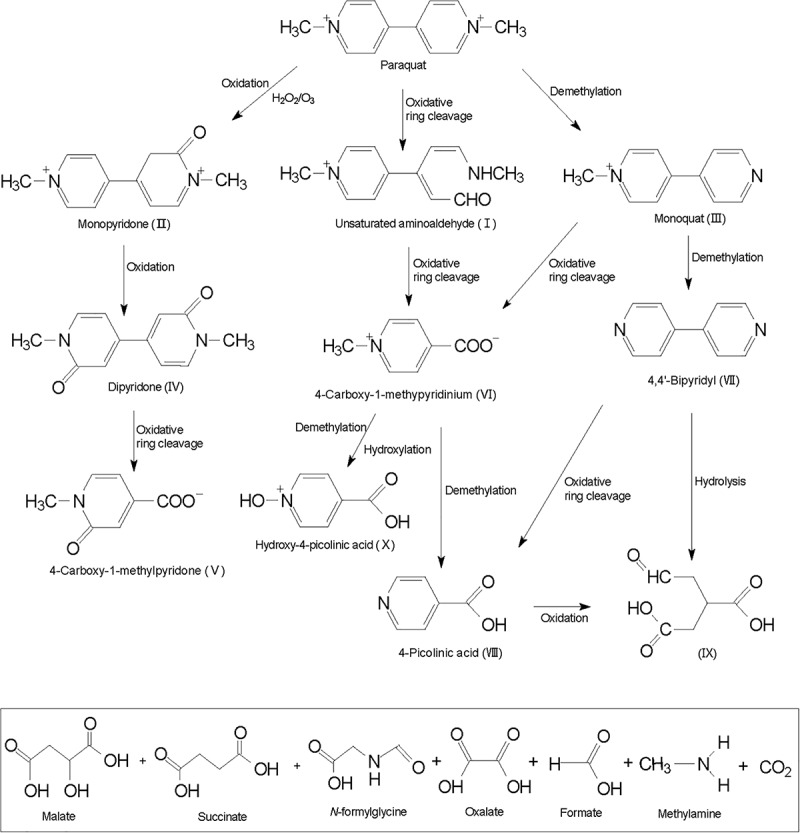
Possible pathways of physicochemical degradation of paraquat.

Hitherto, there is no specific literature about the paraquat degradation pathways and its byproducts. Some studies report monoquat and 4-carboxy-1-methylpyridinium as intermediate products of physiochemical paraquat degradation. Slade (1965) proposed that paraquat degradation is initiated by opening the pyridine ring between nitrogen atoms and adjacent carbon atoms, and unsaturated amino aldehyde (I) is generated through the cleavage of oxidation ring (Figure 2). Kearney et al. (1985) reported that when paraquat reacts with a strong oxidant, such as hydrogen peroxide, monopyridone (II) and monoquat (III) are produced as oxidation and demethylation products. Further oxidation of monopyridone forms dipyridone (IV) and opening up the dipyridone oxidative ring leads to the formation of 4-carboxy-1-methylpyridone (V). Oxidative ring cleavage and demethylation of monoquat leads to the formation of 4-carboxy-1-methylpyridinium ion (VI) and 4,4′-bipyridyl (VII). 4-Picolinic acid (VIII) could arise via demethylation of 4-carboxy-1-methylpyridinium or oxidative ring cleavage of 4,4′-bipyridyl in a series of reactions similar to the formation of 4-carboxy-1-methylpyridinium ion from paraquat.

A possible intermediate C_6_O_5_H_8_ (IX), produced from oxidation and further hydrolysis of 4-picolinic acid or 4,4′-bipyridyl, and a demethylated ring product were identified as hydroxy-4-picolinic acid (X) (Florêncio et al., 2004). Intermediate products do not always completely degrade, and some ring fragmentation products have been identified as malate, succinate, *N*-formylglycine, oxalate, formic, and methylamine (Dhaouadi and Adhoum, 2009). Cantavenera et al. (2007) demonstrated that after TiO_2_-based paraquat degradation and continuous mineralization, nitrate and ammonium ions gradually accumulated and reached up to 83 and 12% of the initial nitrogen concentration in paraquat (Dhaouadi and Adhoum, 2009).

## Microbial Degradation of Paraquat

Bacterial and fungal species belonging to different genera have been isolated from paraquat-contaminated soils by enrichment culture techniques and characterized based on biochemical and molecular tools. Studies have confirmed that some bacterial and fungal (Table 1) species can degrade paraquat in soils and slurry. As illustrated in the table, several degrading microorganisms have been isolated from contaminated soils and deposited to respective microbial culture banks. Four bacterial strains including *Aerobacter aerogenes*, *Agrobacterium tumefaciens*, *Pseudomonas fluorescens*, and *Bacillus cereus* have been characterized for the mineralization of paraquat and can utilize paraquat as a sole growth source of carbon or nitrogen (Tu and Bollen, 2006). Bacteria such as *Oscillospira* sp. BCK-1, *Clostridium prazmowski* BCK-2, and *Sporohalobacter orenetal* BCK-3 efficiently degraded paraquat up to 79.35, 80.26, and 86.22%, respectively, after 3 days of treatment (Han et al., 2014).

**TABLE 1:** Paraquat-degrading strains isolated from various sites and their degradation potential.

**Strains**	**Source**	**Comments**	**References**
*Pseudomonas putida*	Businesses	47.3% of paraquat (69.76 mg/L) was degraded within 3 days in the presence of 25% nutrient About 95% degradation of paraquat (69.76 mg/L) within 3 days in the presence of 15 g/L activated charcoal	Zauscher et al., 2002
*Oscillospira* sp. BCK-1	Paraquat-contaminated soil, China	79.4% degradation of paraquat was achieved after 3 days	Han et al., 2014
*Clostridium prazmowski* BCK-2	Paraquat-contaminated soil, China	80.3% degradation of paraquat was achieved after 3 days	Han et al., 2014
*Sporohalobacter orenetal* BCK-3	Paraquat-contaminated soil, China	86.2% degradation of paraquat was achieved after 3 days	Han et al., 2014
*Corynebacterium fascians* Dows	No data	After 4 weeks training grew in dextrose broth containing 10 000 ppm bipyridylium ion	Tu and Bollen, 2006
*Aerobacter aerogenes*	Soil	Utilize paraquat (10 ppm) as sole carbon and nitrogen source Lag periods of 1–5 days	Tu and Bollen, 2006
*Agrobacterium tumefaciens*	Soil	Utilize paraquat (10 ppm) as sole carbon and nitrogen source Lag periods of 1–5 days	Tu and Bollen, 2006
*Pseudomonas fluorescens*	Soil	Utilize paraquat (10 ppm) as sole carbon and nitrogen source Lag periods of 1–5 days	Tu and Bollen, 2006
*Bacillus cereus*	Soil	Utilize paraquat (10 ppm) as sole carbon and nitrogen source Utilize paraquat as nitrogen source, grew faster than other strains Lag periods of 1–5 days	Tu and Bollen, 2006
*Enterobacter cloacae* PQ02	Paraquat-applied paddy soil, China	About 95% degradation of paraquat (50 mg/L) within 7 days in the presence of extra electron donor such as anthraquinone-2,6-disulfonic acid (AQDS) and sucrose	Wu et al., 2013
*Micrococcus sp. S2*	Soil, Indonesia	About 20% of paraquat (40 mg/L) was degraded within 48 h	Margino et al., 2007
Mixed bacteria (10% *Roseateles terrae* + 25-50% *Bacillus* sp. + 15–35% *Escherichia coli* + 20–50% *Pseudomonas fluorescens*)	Institutes	97% of initial dose (100 mg/L) was degraded after 7 days	Li et al., 2017
*Trametes pavonia* ECS-67	Soil, Mexico	54.2% of paraquat (100 mg/L) was degraded within 12 days Initial biomass concentration of 1 g/L	Camachomorales et al., 2017b
*Trametes versicolor* ECS-79	Soil, Mexico	54.1% of paraquat (100 mg/L) was degraded within 12 days Initial biomass concentration of 1 g/L	Camachomorales et al., 2017b
*Hypholoma dispersum* ECS-705	Soil, Mexico	1. 70.7% of paraquat (100 mg/L) was degraded within 12 days 2. Initial biomass concentration of 1 g/L	Camachomorales et al., 2017b
*Polyporus tricholoma* ECS-58	Soil, Mexico	32% of initial dose (47 mg/L) was removed within 12 days	Camachomorales et al., 2017a
*Cylindrobasidium laeve* ECS-91	Soil, Mexico	26% of initial dose (25 mg/L) was removed within 12 days	Camachomorales et al., 2017a
*Deconica citrispora* ECS-77	Soil, Mexico	47% of initial dose (25 mg/L) was removed within 12 days	Camachomorales et al., 2017a

The biodegradation rate in controlled conditions is influenced by multiple factors including temperature, pH, nutrients, initial concentration, inoculum size and properties of the bacterial or fungal strain (Chen et al., 2015; Cycoń and Piotrowska-Seget, 2016; Zhan et al., 2018b). Zauscher et al. (2002) demonstrated that *Pseudomonas putida* degraded paraquat up to 95% (69.76 mg/L) in the presence of 15 g/L activated charcoal after 3 days of treatment, whereas only 47.3% of paraquat was degraded after substituting activated carbon with nutrients. It has been reported that *Corynebacterium fascians* Dows tolerated extremely high concentrations of bipyridylium ion (10000 mg/L) in dextrose broth up to 4 weeks (Tu and Bollen, 2006). Wu et al. (2013) reported that *Enterobacter cloacae* PQ02 degraded approximately 95% of the initial paraquat dose (50 mg/L) in the presence of extra electron donor anthraquinone-2,6-disulfonic acid (AQDS) and sucrose within 7 days. These carbon sources can easily be utilized by bacteria and accelerate their growth during lag phase. Some studies have revealed that the use of mixed bacterial culture (consortium) resulted in enhanced degradation of pollutants as mixed bacterial culture follows co-metabolism for pollutant degradation (Nawong et al., 2018; Tian et al., 2018). Li et al. (2017) used four microorganisms including *Roseateles terrae*, *Bacillus* sp., *Escherichia coli*, and *P. fluorescens*, in a mixed culture for paraquat degradation, and achieved 97% degradation of initial paraquat dose (100 mg/L) over 7 days. Bacterial strains exhibited significant degradation ability and provided a potential tool for bioremediation of paraquat-contaminated environments.

Besides bacteria, fungal systems can also effectively degrade paraquat. *Lipomyces starkeyi* Lod and Rij completely removed paraquat (27 mg/L) from the medium within 3 days. However, when the paraquat concentration was increased twofold (54 mg/L), biomass and paraquat degradation notably decreased to less than 10% (Alexander, 1999). It was noticed that *L. starkeyi* could degrade paraquat under aerobic conditions. Biodegradation studies revealed that paraquat-degrading microorganisms do not exhibit similar efficiency in degrading different concentrations of paraquat. Camachomorales et al. (2017b) isolated 54 macromycetes from southeastern Mexico, and only three (*Trametes pavonia* ECS-67, *Trametes versicolor* ECS-79, and *H. dispersum* ECS-705) presented 54.2, 54.1, and 70.7% of paraquat (100 mg/L) degradation within 12 days. In another study Camachomorales et al. (2017a) revealed that three other macromycetes including *Polyporus tricholoma* ECS-58 (32%, 75 mg/L), *Cylindrobasidium laeve* ECS-91 (26%, 25 mg/L), and *Deconica citrispora* ECS-77 (47%, 25 mg/L), showed lower paraquat degradation after 12 days of incubation.

**FIGURE 3:**
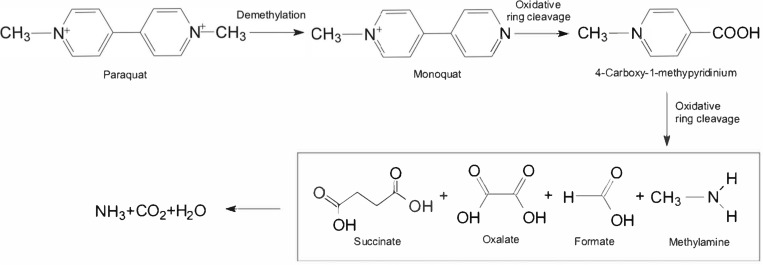
Metabolic pathways of paraquat in microorganisms.

To date, degradation pathways of paraquat in microorganisms have never been reported. As shown in Figure 3, the 1st step in paraquat conversion is demethylation to form monoquat through microbial activity. In the next step, further oxidative ring cleavage of monoquat forms 4-carboxy-1-methylpyridinium ion (Dinis-Oliveira et al., 2008). Pyridinium ring carbons are released as CO_2_ by ^14^C-labeling, and 4-carboxy-1-methylpyridinium ion readily degrades in soils into methylamine and CO_2_ by microbial activity (Singh and Singh, 2016). Methylamine can be used as a source of nitrogen and carbon for microbial growth. During the paraquat biodegradation, ring fragmentation products identified as three carboxylic acids were oxalate, formate and succinate whereas methylamine and carbon dioxide were detected as the ultimate metabolites (Dinis-Oliveira et al., 2008). However, the enzymology of paraquat degradation is rarely reported and other intermediates have not been identified. Paraquat-degrading microbes might utilize the product from upstream pathways of cell energy via glycolytic and tricarboxylic acid pathways. Further studies about paraquat biodegradation are required to detect common metabolites and enzymes responsible for converting different intermediates.

## Bioremediation Potential of Paraquat-Degrading Microorganisms

Microbial remediation is the process of transforming highly toxic compounds into low-toxic or non-toxic products after a series of domestication, enrichment, screening and culturing of the strains having degradation characteristics (Chen et al., 2015; Liu et al., 2015; Cycoń and Piotrowska-Seget, 2016). Bioremediation is supposed to be more promising for the removal of chemical pollutants in water and soil environments (Chen et al., 2014; Yang et al., 2018). Physicochemical methods of controlling or mitigating environmental pollution were less effective and more expensive than biological methods of remediation (Arora et al., 2018). The use of microorganisms for bioremediation of contaminated sites may be a viable alternative to conventional clean-up methods because a variety of microorganisms are known to utilize chemical pollutants as a sole carbon or energy source (Gonzalez-Marquez et al., 2019). In agriculture, paraquat residues in the topsoil layer and plant surface are degraded by photolysis, while most of the remaining are absorbed by clay lattice and loose weeding activity. According to Alexander (1999), paraquat is completely degraded by soil microorganisms within 6 years into ammonia, carbon dioxide and water (Figure 3). Hence, microbial bioremediation is considered as an efficient, safe and cost-effective strategy to remove paraquat from contaminated environments. Most of the microorganisms in the environment (90∼99%) cannot be cultured, and paraquat half-life in the soil can be as long as 6.6 years (Alexander, 1999). Therefore, it is necessary to isolate and identify the high-efficiency paraquat-degrading microorganisms and determine their potential for the bioremediation of paraquat-contaminated environments. To solve isolation difficulties, metagenomics-based approaches can be followed for paraquat biodegradation study.

A few paraquat-degrading strains have been isolated, but their degradation efficiency in fields is unstable. Response surface method can optimize the conditions for microbial degradation of pollutants (Chen et al., 2013; Xiao et al., 2015). Hitherto, studies involved in paraquat-degrading microbes are few, and mainly include bacteria. The paraquat degradation rate can nearly reach 100% after adding exogenous electron donor or activated carbon. For example, *P. putida* and *E. cloacae* PQ02 showed a 95% paraquat degradation rate after the addition of activated carbon and glucose. It is worth mentioning that in a patent filed by Li et al. (2017), a bacterial consortium achieved 97% paraquat degradation (100 mg/L). There is less information about the fungal degradation of paraquat, and the overall degradation effect of fungi is not as significant as that of bacteria. A recent study on fungi by Camachomorales et al. (2017b) indicated that 70.7% paraquat was removed by *Hypholoma dispersum* ECS-705 in 12 days. Bacteria metabolize paraquat in two ways: (1) bacteria utilize paraquat as the sole nitrogen or carbon source, and (2) bacteria transform paraquat into low-toxic or non-toxic products through co-metabolism. In both cases, microbial biodegradation of paraquat is quite promising.

## Conclusion and Future Perspectives

Large-scale paraquat applications in the agriculture sector are urgently demanded to mitigate the effects of this compound. Established hazardous impacts of paraquat on humans and environment urge us to develop safe, efficient and economical technologies for the remediation of paraquat-contaminated environments. During the last two decades, physicochemical degradation based on AOPs has been developed as an effective measure to remove paraquat residues from sewage. However, considering the high cost and uncontrollable reaction conditions, it is not widely applicable.

Bacteria isolated from paraquat-contaminated environments with high degradation capacity and potential for bioremediation are considered as the most promising strategy. However, the published literature generally reveals the use of a single strain for bioremediation testing, which could not produce ideal effects under field conditions. To overcome such problems, consortia consisting of various bacteria could be used for large-scale applications. Members of consortia play various roles in different stages of degradation and can produce better degradation effects than a single strain.

In order to achieve higher degradation efficiency, the relationship between members of such a consortium and their adaptability to adverse environments should be studied. In addition, it is important to screen degrading bacteria that could sustain a wide range of soil environmental factors such as pH, temperature, salinity, heavy metals, and nutrient availability.

The role of functional genes and enzymes in bioremediation of paraquat-contaminated environment is the key to understand degradation mechanism of paraquat. Although many paraquat-degrading microorganisms have been isolated and characterized in previous studies, their functional genes have not yet been reported. Therefore, further studies about related functional genes and enzymes are needed before the field-scale applications of paraquat-degrading microorganisms. Modern high-throughput omics technologies can facilitate to achieve clear information about the metabolic pathway, regulatory genes and enzymes for paraquat biodegradation.

## Author Contributions

SC conceived the idea. YH contributed to the writing and prepared the figures and tables. HZ, PB, and SC participated in revising the manuscript. All authors approved the final manuscript for publication.

## Conflict of Interest Statement

The authors declare that the research was conducted in the absence of any commercial or financial relationships that could be construed as a potential conflict of interest.
